# Warranting Dental Operations

**Published:** 1862-04

**Authors:** J. Taft


### WARRANTING DENTAL OPERATIONS.

BY J. TAFT.

THE practice of warranting dental operations has been so general, that almost every one expects it; and many require a guaranty as a condition in permitting the dentist to operate. This has arisen from several circumstances. The disposition to make what they suppose a "good bargain" exists in a very strong degree in many persons. There is a very general opinion that dental operations, especially those upon the teeth, are of short duration and frequently inefficient, and that it is only a matter of compliment to the dentist that he is permitted to operate at all. Then, again, the dentists are as much at fault in this matter as their patients, for they in too many cases volunteer the guaranty unasked for, and always yield to it when required, in words at least. The present attainments of the profession enable those who are well prepared to attain as efficient results as those of any other department of medical practice, and the dental practitioner should no more be required to give a guaranty for the efficiency of his treatment than he who treats a fever, or amputates a limb; either may be badly done, but a thousand guaranties would not atone for bad treatment in either case. No one thinks of calling upon a physician and requiring of him a guaranty to cure, as a condition of his treating the case, nor a warrant that the patient will not become sick again after he is once cured. No man, who is not an empyric, would practice under such requirements. There is no more justice in requiring the *dentist* to warrant his operations than the physician his treatment.

If the dentist performs his operations as perfectly as the present attainments of the profession will enable him, and his fee for it is just, then no one has a right to demand, or even require that he should perform the same amount of labor, and be at the same expense again without compensation. But the reply is often made, "It was not well done at first, and the dentist should make his work good." This is a presumption by the patient that the operation in question was not well done. Many things that are well done, and among these some operations upon the teeth, will not necessarily be of long duration. The circumstances are sometimes such as to preclude permanency, at least in an extended sense. This may result from the character of the teeth themselves, and sometimes from the neglect of the patient, and sometimes from the great difficulty of making a good operation, because of interruptions and difficulties, such as a contracted mouth, cavities badly located, an abundant and uncontrollable flow of saliva, and great irritability and restlessness of the patient.

The presumption is that every dentist performs his operations as well as he can at the time and under the circumstances—perhaps not as well as he might, were he to avail himself of greater facilities. Again, a portion in some teeth—some part of the walls of a cavity—may be quite thin, so that it would be comparatively easily broken, which, if cut away, would to some extent mar the beauty of the tooth. The patient and operator both concur in an effort to preserve such part of the tooth, by filling the cavity, and thus sustaining the frail part. Any one can readily understand that in such a case, the desire to preserve the beauty of a tooth will lead an operator occasionally to place reliance where it will not be realized, the tooth become broken, the filling exposed and displaced. Some of the finest and most perfect fillings we ever saw, in regard to execution, were lost just in this way.

Now, such cases are much more difficult to fill, and require more skill, than if such thin portion were cut away; now since the operator is expending the greater skill, time and labor to obtain the highest possible good for the patient, is it right that, in case of failure, the operator should suffer the loss? How came the tooth so much decayed, that the cavity is large and its borders thin and friable? It is by the neglect of the patient to give attention to decay in its early stages, and it is but just that he should be made to pay the penalty of his neglect ; nature will demand it, and make him pay it, too.

No one should employ a dentist in whom he has not full confidence,—believe that he can do as well as can be done under the circumstances,—and if the operator does this, justice does not require that he should be responsible for subsequent accidents.

There are cases in which no operation for the preservation of the natural teeth will be of long duration, but is at *best* only temporary, owing to the defective structure of the teeth. In all such cases, however, the dentist should give his patient the most perfect understanding of the nature of the case, and the probabilities concerning it ; he should always impress upon the mind of his patient that much depends upon his own care and effort for the future preservation of his teeth, however perfectly they may have been treated by the dentist.

The ordinary course pursued by those who have had teeth filled, and permanency guaranteed, is to neglect them ; they think and often say, that their teeth are filled and warranted, and the responsibility is all upon the dentist, and that it is unnecessary for them to spend time and labor upon them ; that if the teeth decay and the plugs come out, the dentist is pledged to fill them again without charge. We have seen persons so neglect their teeth, after they were filled and put in good condition, that they became invested, to a very great extent, with salivary calculus, and a thick, gummy mucus, that rendered them disgusting to both sight and smell,—fillings that had been introduced were entirely covered. We have known a few just such cases, in which the fillings were lost, and the patients would have the assurance to return and require that they be filled without charge.

We would advise that the teeth of such persons be not touched a second time, even though the patient be willing to pay a fee, till he shall go and do works that shall evidence repentance.

Every dentist either performs his operations as well as he can, under the circumstances in each case, or he does not; if his best and first efforts are inefficient, second or third efforts of a similar character will be worthless, though made in fulfillment of a guaranty; for the more frequent an imperfect operation is performed upon a tooth, the more difficult the case becomes, and the less probability is there of ultimate success. And if the operator does not perform his work well in the first effort, when under a guaranty and the influence of a prospective fee, is it probable he will do better in a second effort, when there is neither compensation nor pledge?

The fees of the dentist are not estimated or governed by the value accruing to the patient, by virtue of the operation —for this is past reckoning—but they are governed by the required skill, labor, time and expense of the dentist. This alone can be the basis of his fees. The patient, for good operations upon the natural teeth, receives ten to a hundred fold the value of any ordinary fee charged.

This whole thing of guaranteeing dental operations originated in, and savors of, quackery; it originated with, and is continued by, those who have not a sufficient measure of professional attainment upon which to base a reputation. No man who *knows* that his operations, under all favorable circumstances, will be efficient, and that all his operations are as good as the present status of the profession will allow, will degrade himself by warranting them. This practice springs from a consciousness that the work will require to be done again.

